# Cross-Sectional Study of Sleep Quantity and Quality and Amnestic and Non-Amnestic Cognitive Function in an Ageing Population: The English Longitudinal Study of Ageing (ELSA)

**DOI:** 10.1371/journal.pone.0100991

**Published:** 2014-06-26

**Authors:** Michelle A. Miller, Hayley Wright, Chen Ji, Francesco P. Cappuccio

**Affiliations:** University of Warwick, Warwick Medical School, Division of Mental Health and Wellbeing, Coventry, United Kingdom; Cardiff University, United Kingdom

## Abstract

**Background:**

The aim was to investigate the association between sleep disturbances and cognitive function in younger and older individuals from an ageing population.

**Methods:**

3,968 male and 4,821 female white participants, aged 50 years and over, from the English Longitudinal Study of Ageing (ELSA) were studied. Information on sleep quality and quantity as well as both amnestic (memory, ACF) and non-amnestic (non-memory, nACF) function was available at Wave 4 (2008). Analysis of covariance was used to evaluate the relationship between sleep and cognitive function.

**Results:**

After adjustment for multiple confounders in the younger group (50–64 years) duration of sleep explained 15.2% of the variance in ACF (*p* = 0.003) and 20.6% of nACF (*p* = 0.010). In the older group (65+ years) the estimates were 21.3% (*p*<0.001) and 25.6% (*p*<0.001), respectively. For sleep quality, there was a statistically significant association between sleep quality and both ACF (*p*<0.001) and nACF (*p*<0.001) in the older age group, but not in the younger age group (*p* = 0.586 and *p* = 0.373, respectively; interaction between age and sleep quality in the study sample including both age groups: *p*<0.001 for ACF and *p* = 0.018 for nACF). Sleep quality explained between 15.1% and 25.5% of the variance in cognition. The interaction with age was independent of duration of sleep. At any level of sleep duration there was a steeper association between sleep quality and ACF in the older than the younger group.

**Conclusions:**

The associations between sleep disturbances and cognitive function vary between younger and older adults. Prospective studies will determine the temporal relationships between sleep disturbances and changes in cognition in different age groups.

## Introduction

Developments in the fields of sleep neurobiology and cognitive neuroscience have produced converging evidence of a fundamental role for sleep in cognition. As we age the amount of time spent in good quality, continuous sleep decreases [1] and there are links between sleep disturbances and cognitive impairment in vulnerable populations, such as those at risk of various dementing illnesses [2], [3]. Sleep disturbances, including specific reduction in fast sleep spindles, may be one of the earliest signs of neurodegenerative disorders, including early Alzheimer’s disease (AD) [4].

The term ‘cognition’ refers to a range of mental processes including memory, problem solving, language, forward planning and attention, which can all be differentially affected by inadequate sleep. Specific cognitive processes can be grouped into two broader categories of amnestic function (ACF, referring to memory) and non-amnestic function (nACF, or non-memory). This dichotomy is of particular importance in relation to the progression from normal cognitive ageing to mild cognitive impairment (MCI), since MCI is typically diagnosed as amnestic (aMCI) or non-amnestic (naMCI) subtype [5]. These two subtypes of MCI may possibly have different trajectories, where aMCI may potentially develop into AD, and naMCI may develop into other forms of dementia (e.g. vascular dementia, Dementia with Lewy bodies (DLB), fronto-temporal dementia) [6], [7], although the validity of this prediction has previously been questioned [8], [9].

In cross-sectional studies both short and long sleep durations are associated with diminished global cognition [10] and memory function [11] in adult populations. However, whilst there may be different associations between sleep disturbances and subtypes of MCI [12], most studies have reported the effects of sleep on global cognition, using generalised tests such as the Mini-Mental State Exam (MMSE) [13].

A recent study [14] attempted to distinguish between amnestic and non-amnestic cognitive impairments with relation to sleep patterns. The specific amnestic cognitive impairments were identified however, simply by using the scores on the delayed recall task from the MMSE.

Amongst women, long sleep (>9 hr per night) predicted cognitive impairment over one year, whilst in men, it was short sleep (<5 hr per night) that predicted cognitive decline. In a separate study, disturbed sleep was strongly associated with a decline in executive (non-amnestic) function but less so for global cognition [15].

The current study explores the associations between measures of sleep and amnestic and non-amnestic cognitive function in younger and older adults in a representative population of English men and women over the age of 50 years.

## Methods

### Study Population

The English Longitudinal Study of Ageing (ELSA) is a representative sample of the English population aged 50 years and over (*N* = 11,050) [16], [17]. Data were selected from Wave 4 (2008), at which point sleep data was included for the first time, alongside routine measures of health, disease, cognition, finances, lifestyle and anthropometrics. The full methodology, sampling procedures and details on previous waves of screening have been reported elsewhere [18]. Non-whites (3.5%) and participants under the age of 50 (n = 301) or aged 90 years or above (n = 137) were excluded, as were those with inaccurate or incomplete essential data (n = 1432). The remaining subjects with full data on sleep quantity and quality and cognitive function were included (n = 8,789; 3,968 males and 4,821 females) [18]. Since research has shown pre- and post-retirement changes in health conditions [19] and sleep behaviour [20], the main objective of our analysis was to explore the patterns of associations between sleep and cognition at different stages of ageing. As significant interactions were detected between sleep disturbances and age (quantity: *p*<0.001 for ACF and *p* = 0.06 for nACF; quality: *p*<0.001 for ACF and *p* = 0.018 for nACF), the respondents were separated into younger (50–64 years; n = 4,660) and older (65+ years; n = 4,129) age groups and analyses carried out separately.

### Ethics statement

All participants of ELSA provided signed consent, and ethical approval was granted by the London Multi- Centre Research Ethics Committee. Anonymised unlinked data for this secondary data analysis initiative study was provided by the UK Data Service (previously known as Economic and Social Data Service (ESDS)).

### Measures of Exposure

#### Sleep Quantity

Respondents reported the number of hours they slept, on average, per weeknight. Open-ended responses ranged between 3 and 14 hours, and were categorised as <6 hrs, 6–8 hrs, and >8 hrs for comparability with previous publications [21], [22], whilst maintaining adequate numbers in each group. Throughout the description of results, we will refer to the <6 hrs group as ‘short sleepers’, the 6–8 hrs group as ‘optimal sleepers’ [23], and the >8 hrs group as ‘long sleepers’.

#### Sleep Quality

Respondents reported the frequency with which they experienced the following over the previous month: delay in falling asleep, inability to stay asleep, and waking up feeling tired. The response categories were: ‘no difficulties’, ‘less than once a week’, ‘once or twice a week’, and ‘three times or more a week’, and were assigned a numerical score from 1 to 4. An overall rating of sleep quality over the previous month, ranging from ‘very good’, ‘good’, ‘fairly bad’, to ‘very bad’, was assigned a numerical score of 1 to 4. All scores were summed and then categorised into tertiles, with the first tertile representing those reporting the least sleep disturbance (lowest scores), and the third tertile those reporting the most sleep disturbance (highest scores).

### Measures of Outcome

#### Amnestic tasks

Tests assessing amnestic function in ELSA consist of a prospective memory task (remembering to carry out a task at a specified time during the test; range 0–3), orientation questions (reporting the day, date, month and year; range 0–4), and immediate and delayed recall tasks (memory for a list of 10 everyday words; each range 0–10). Details of each amnestic test have been described in detail elsewhere [24].

#### Non-amnestic tasks

The non-amnestic tests in ELSA consist of verbal fluency (number of animals named in one minute; range 1–55), speed of processing (total number of letters searched in one minute range 34–780), visual search accuracy (percentage of target letters detected in one minute; range 0–100) [24], and numeracy (mental arithmetic questions of increasing difficulty; range 0–6) [25].

Raw scores were standardised to allow meaningful comparisons between scores and to allow combined scores to be calculated, with standardisation being performed separately for each age group. Within each age group, each cognitive test score was converted to a z score using the standard formula (where *x* = raw score; *x–* = group mean; *s* = standard deviation):
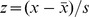



For ease of interpretation and comparison with previous studies [26] z scores were converted to T scores using the standard formula:
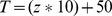



The same procedure of converting raw scores into z scores, and then z scores into T scores, was repeated for each cognitive test. To calculate an average T score across amnestic tests for each respondent, T scores for prospective memory, date questions, immediate recall and delayed recall were summed and divided by 4. Likewise, to calculate an average T score across non-amnestic tests for each respondent, T scores for verbal fluency, speed of processing, search accuracy and numeracy were summed and divided by 4.

### Measures of Covariates

Standard adjustments were made for age and sex, where age was measured in full years and entered into the models as a continuous variable. The effects of further covariates known to influence sleep and/or cognition were assessed at each stage of the analysis, although not all were included in the final models. These covariates were all derived from the questionnaire administered in ELSA Wave 4, and were as follows: highest educational qualification (none, intermediate, or degree/higher; [27]); employment grade (managerial/professional, intermediate, or routine/manual; [26]); marital status (single, married, separated/divorced, or widowed; [27]); depression (categorised as either ‘depressed’, where CES-D score ≥4 [28], [29], or there had been a previous diagnosis of depression; or ‘not depressed’, where CES-D score <4 and there was no reported diagnosis of depression); quality of life (total CASP-19 score; [30]); physical activity (sedentary, low, moderate, or high; composite measure derived from four questions in the main ELSA interview, approximating closely to the classification used in the Allied Dunbar Survey of Fitness [31]); smoking (never, current smoker, or previous smoker; [32]); alcohol consumption (units per week); chronic disease (reported diagnosis of one or more of the following cardiovascular, chronic or respiratory complaints; high blood pressure, angina, myocardial infarction, congestive heart failure, heart murmur, arrhythmia, diabetes or high blood sugar, stroke, high cholesterol, arthritis, osteoporosis, cancer, Parkinson’s Disease, psychiatric disease, Alzheimer’s Disease, dementia or memory impairment, lung disease i.e. chronic bronchitis or emphysema, or asthma); limiting longstanding illness (any self-reported illness, disability or infirmity which has limited activity over a period of time); troubled by pain; and finally, self-reported general health (excellent, very good, good, fair or poor).

### Statistical Analysis

Age and basic characteristics were derived from the ELSA questionnaire at Wave 4, where chi-square and one-way analysis of variance (ANOVA) were used to determine differences in the distribution of variables. Analysis of covariance (ANCOVA) was performed to test the association between sleep quantity and sleep quality, and scores of amnestic and non-amnestic cognition separately, whilst controlling for the effects of confounding/contributing factors. ANCOVAs were carried out separately for sleep quantity and sleep quality, for amnestic and non-amnestic scores, and for each age group, although the final models for each analysis were the same. We tested for interactions between each of the sleep factors and each covariate separately, with the intention to include any interactions in the final models. There were interactions between the sleep variables and age at this level for both the ACF and the nACF (*p*<0.001 and *p* = 0.06 with sleep quantity and *p*<0.001 and *p* = 0.018 for sleep quality, respectively). The final models included either average amnestic or non-amnestic T scores as the dependant variable (measure of outcome), either sleep quantity categories or sleep quality tertiles as the independent variables (measure of exposure), and all of the following covariates: age, sex, education, employment grade, depression, physical activity, smoking, general health, plus a sleep*age interaction term. A two-sided *p* value <0.05 is considered statistically significant. All analyses were performed in IBM SPSS Statistics version 21 (IBM Corp, Armonk, NY).

## Results

### Population characteristics

The basic characteristics and demographics of the study sample are presented by sleep quantity categories (Table 1) and sleep quality tertiles (Table 2), in younger (50–64 years; n = 4,660) and older (65+ years; n = 4,129) age groups.

**Table 1:** Characteristics of study population by sleep QUANTITY categories.

	YOUNGER (50–64 years)				OLDER (65+ years)			
Variable^1^	<6 hr	6–8 hr	>8 hr	*p* 2	<6 hr	6–8 hr	>8 hr	*p* 2
***N*** ** [%]** unless otherwise stated	646[13.9]	3778[81.1]	236[5.1]		607[14.7]	3136[76.0]	386[9.3]	
**Sex (% male)**	37.3	46.2	38.6	<0.001	37.4	47.5	45.3	<0.001
**Age (years)**	57.9 (3.8)	57.9 (3.8)	58.3 (4.2)	= 0.252	73.9 (6.1)	73.2 (6.1)	74.5 (6.5)	<0.001
**Amnestic function (T score)**	48.4 (6.3)	50.4 (6.2)	48.6 (6.9)	<0.001	49.4 (6.6)	50.4 (6.7)	47.5 (8.4)	<0.001
**Non-amnestic function (T score)**	48.8 (5.5)	50.3 (5.4)	48.4 (5.8)	<0.001	49.2 (5.7)	50.4 (6.0)	48.3 (6.2)	<0.001
**Educational Qualification:**	**[** ***N*** ** = 610]**	**[** ***N*** ** = 3577]**	**[** ***N*** ** = 226]**		**[** ***N*** ** = 605]**	**[** ***N*** ** = 3122]**	**[** ***N*** ** = 385]**	
None	26.1	15.5	28.8		44.6	34.7	37.7	
Intermediate	45.6	41.8	42.5		35.0	36.7	37.1	
Higher/degree	28.4	42.6	28.8	<0.001	20.3	28.6	25.2	<0.001
**Employment Grade:**	**[** ***N*** ** = 611]**	**[** ***N*** ** = 3594]**	**[** ***N*** ** = 227]**		**[** ***N*** ** = 597]**	**[** ***N*** ** = 3077]**	**[** ***N*** ** = 376]**	
Managerial/Professional	29.0	39.8	31.7		22.6	32.9	25.3	
Intermediate	24.5	25.8	23.8		25.5	26.3	26.1	
Routine/Manual	46.5	34.4	44.5	<0.001	51.9	40.9	48.7	<0.001
**Marital Status:**								
Single	6.3	6.9	5.1		5.3	4.4	4.7	
Married	66.1	76.0	78.8		54.7	63.7	58.3	
Divorced/Separated	20.7	13.2	13.1		9.4	8.6	4.4	
Widowed	6.8	3.9	3.0	<0.001	30.6	23.2	32.6	<0.001
**Depression** 3 **:**	**[** ***N*** ** = 645]**	**[** ***N*** ** = 3775]**	**[** ***N*** ** = 236]**		**[** ***N*** ** = 607]**	**[** ***N*** ** = 3130]**	**[** ***N*** ** = 385]**	
Yes	53.0	22.6	33.9	<0.001	51.1	26.9	30.1	<0.001
**Quality of Life:**	**[** ***N*** ** = 565]**	**[** ***N*** ** = 3345]**	**[** ***N*** ** = 202]**		**[** ***N*** ** = 494]**	**[** ***N*** ** = 2668]**	**[** ***N*** ** = 304]**	
CASP19 score	37.4 (10.1)	42.4 (8.4)	40.7 (9.2)	<0.001	37.1 (9.0)	41.4 (7.9)	40.0 (8.7)	<0.001
**Physical Activity level:**	**[** ***N*** ** = 645]**	**[** ***N*** ** = 3777]**	**[** ***N*** ** = 236]**		**[** ***N*** ** = 607]**	**[** ***N*** ** = 3134]**	**[** ***N*** ** = 386]**	
Sedentary	5.6	2.0	5.1		12.0	7.7	10.4	
Low	25.0	16.5	22.9		35.6	26.3	31.1	
Moderate	48.4	53.8	51.3		40.9	49.5	46.1	
High	21.1	27.6	20.8	<0.001	11.5	16.5	12.4	<0.001
**Smoking status:**								
Never smoked	62.1	70.5	67.4		78.4	81.1	78.0	
Current smoker	19.8	17.3	17.8		12.2	10.7	10.4	
Previous smoker	18.1	12.2	14.8	<0.001	9.4	8.3	11.7	= 0.152
**Alcohol consumption:**	**[** ***N*** ** = 325]**	**[** ***N*** ** = 2548]**	**[** ***N*** ** = 145]**		**[** ***N*** ** = 269]**	**[** ***N*** ** = 1797]**	**[** ***N*** ** = 177]**	
Units per week	16.8 (17.2)	17.8 (16.3)	17.8 (16.7)	= 0.436	14.2 (15.9)	16.0 (15.6)	13.0 (11.8)	= 0.013
**Diagnosed disease** 3	82.2	69.2	78.8	<0.001	89.1	87.7	86.8	= 0.491
**Limiting longstanding illness** 3	46.4	24.1	38.6	<0.001	49.9	35.5	42.7	<0.001
**Troubled by pain** 3	57.9	34.3	41.1	<0.001	56.8	38.5	36.3	<0.001
**Self-reported general health:**								
Excellent	10.5	18.0	11.4		6.1	10.0	7.0	
Very good	20.5	34.5	28.0		18.8	27.8	27.5	
Good	28.4	30.4	33.5		30.6	35.2	33.7	
Fair	25.9	13.4	17.8		28.3	20.6	23.1	
Poor	14.7	3.8	9.3	<0.001	16.1	6.3	8.8	<0.001

1 Results expressed as mean (sd) or %.

2 ANOVA for continuous data, chi-square for categorical data (where *p* value represents differences between all categories).

3 See Methods section for description of variables.

**Table 2:** Characteristics of study population by sleep QUALITY^1^ tertiles.

	YOUNGER (50–64 years)				OLDER (65+ years)			
Variable^2^	1st	2nd	3rd	*p* 3	1st	2nd	3rd	*P* 3
***N*** ** [%]** unless otherwise stated	1714[36.8]	1487[31.9]	1459[31.3]		1573[38.1]	1424[34.5]	1132[27.4]	
**Sex (% male)**	53.9	45.6	32.6	<0.001	52.4	47.8	34.1	<0.001
**Age (years)**	57.8 (3.8)	58.2 (3.8)	57.8 (3.8)	= 0.001	73.3 (6.3)	73.4 (6.0)	73.5 (6.1)	= 0.610
**Amnestic function (T score)**	50.3 (6.3)	50.2 (6.1)	49.4 (6.4)	<0.001	49.7 (7.3)	50.5 (6.7)	49.8 (6.7)	= 0.002
**Non-amnestic function (T score)**	50.5 (5.6)	50.3 (5.3)	49.1 (5.5)	<0.001	50.1 (6.2)	50.4 (5.8)	49.4 (5.9)	<0.001
**Educational Qualification:**	**[** ***N*** ** = 1610]**	**[** ***N*** ** = 1418]**	**[** ***N*** ** = 1385]**		**[** ***N*** ** = 1567]**	**[** ***N*** ** = 1421]**	**[** ***N*** ** = 1124]**	
None	14.7	15.2	23.6		33.8	33.2	44.1	
Intermediate	39.7	44.2	43.6		36.1	38.4	34.8	
Higher/degree	45.6	40.6	32.8	<0.001	30.1	28.4	21.1	<0.001
**Employment Grade:**	**[** ***N*** ** = 1630]**	**[** ***N*** ** = 1419]**	**[** ***N*** ** = 1383]**		**[** ***N*** ** = 1547]**	**[** ***N*** ** = 1398]**	**[** ***N*** ** = 1105]**	
Managerial/Professional	42.9	37.8	32.0		32.1	32.5	26.2	
Intermediate	24.8	25.4	26.5		27.0	26.0	25.1	
Routine/Manual	32.3	36.9	41.4	<0.001	40.9	41.5	48.8	<0.001
**Marital Status:**								
Single	6.9	6.0	7.4		4.5	4.6	4.5	
Married	77.6	75.9	70.3		64.5	62.2	58.0	
Divorced/Separated	12.3	13.7	17.1		7.2	9.1	8.9	
Widowed	3.3	4.4	5.2	<0.001	23.8	24.0	28.6	= 0.016
**Depression** 4 **:**	**[** ***N*** ** = 1711]**	**[** ***N*** ** = 1487]**	**[** ***N*** ** = 1458]**		**[** ***N*** ** = 1570]**	**[** ***N*** ** = 1421]**	**[** ***N*** ** = 1131]**	
Yes	9.6	21.5	54.1	<0.001	15.7	26.9	56.5	<0.001
**Quality of Life:**	**[** ***N*** ** = 1526]**	**[** ***N*** ** = 1319]**	**[** ***N*** ** = 1267]**		**[** ***N*** ** = 1339]**	**[** ***N*** ** = 1197]**	**[** ***N*** ** = 930]**	
CASP19 score	44.6 (7.7)	42.3 (7.9)	37.3 (9.6)	<0.001	43.3 (7.5)	40.6 (7.5)	36.7 (8.8)	<0.001
**Physical Activity level:**	**[** ***N*** ** = 1713]**	**[** ***N*** ** = 1487]**	**[** ***N*** ** = 1458]**		**[** ***N*** ** = 1573]**	**[** ***N*** ** = 1424]**	**[** ***N*** ** = 1130]**	
Sedentary	2.3	1.5	4.3		6.7	7.0	13.1	
Low	12.7	16.0	26.3		23.5	27.2	35.8	
Moderate	55.0	53.4	50.0		51.9	49.5	40.4	
High	29.9	29.1	19.4	<0.001	17.9	16.3	10.7	<0.001
**Smoking status:**								
Never smoked	70.5	71.9	64.8		79.8	82.3	78.7	
Current smoker	17.5	15.8	19.9		11.4	10.0	11.2	
Previous smoker	12.0	12.3	15.4	<0.001	8.8	7.7	10.1	= 0.157
**Alcohol consumption:**	**[** ***N*** ** = 1197]**	**[** ***N*** ** = 1000]**	**[** ***N*** ** = 821]**		**[** ***N*** ** = 910]**	**[** ***N*** ** = 798]**	**[** ***N*** ** = 535]**	
Units per week	18.6 (17.2)	18.4 (17.3)	15.8 (15.2)	= 0.001	16.4 (16.1)	15.8 (15.4)	13.6 (14.2)	= 0.003
**Diagnosed disease** 4	62.3	71.8	82.0	<0.001	83.9	88.4	92.4	<0.001
**Limiting longstanding illness** 4	16.1	24.4	45.4	<0.001	26.3	37.5	56.1	<0.001
**Troubled by pain** 4	23.0	36.5	56.7	<0.001	26.3	40.7	61.6	<0.001
**Self-reported general health:**								
Excellent	24.8	15.7	8.0		13.7	8.3	4.1	
Very good	38.0	34.7	22.8		35.7	25.0	15.3	
Good	27.2	32.3	31.7		34.1	37.3	31.2	
Fair	8.6	13.7	24.9		13.2	23.3	32.6	
Poor	1.3	3.6	12.7	<0.001	3.4	6.1	16.9	<0.001

1 Total sleep quality score divided into tertiles. 1^st^ tertile = least disturbance; 3^rd^ tertile = most disturbance. See Methods for further details.

2 Results expressed as mean (sd) or %.

3 ANOVA for continuous data, chi-square for categorical data (where *p* value represents differences between all categories).

4 See Methods section for description of variables.

Many of the measured variables showed significant differences across categories of sleep quantity or sleep quality in both younger and older age groups. As in previous studies, the proportion of individuals who were long sleepers increased in the older age group; in both age groups there was a strong association between short sleep and depression, reduced quality of life, limiting long standing illness, troubled by pain and poorer self-reported general health. Coexisting diagnosed disease was associated with short sleep in the younger, but not in the older, age group, probably due to the high prevalence of diagnosed disease in the latter (Table 1). For sleep quality, in both age groups, individuals were more likely to have good quality sleep; there were however strong associations between poor quality sleep and depression, reduced quality of life, reduced physical activity, coexisting diagnosed disease, limiting longstanding illness, troubled by pain and poorer self-reported general health (Table 2).

### Sleep quantity, sleep quality and cognitive function

#### Unadjusted

The association between sleep domains (quantity and quality) are shown in Figure S1 (Supplementary Information). In both the younger and older age groups, short sleepers reported the more sleep disturbance. There was a significant variation (inverted U shape) in both amnestic and non-amnestic cognition by sleep quantity in both the younger and older age groups (all p<0.001), whereby cognition scores were lower in both short and long sleepers (Table 1). Likewise for sleep quality, there was a significant variation in amnestic function for younger (p<0.001) and older (p = 0.002) groups, as well as in non-amnestic cognition (both p<0.001), whereby lower cognition scores were associated with poorer quality (Table 2).

#### Adjusted

**Figure 1:**
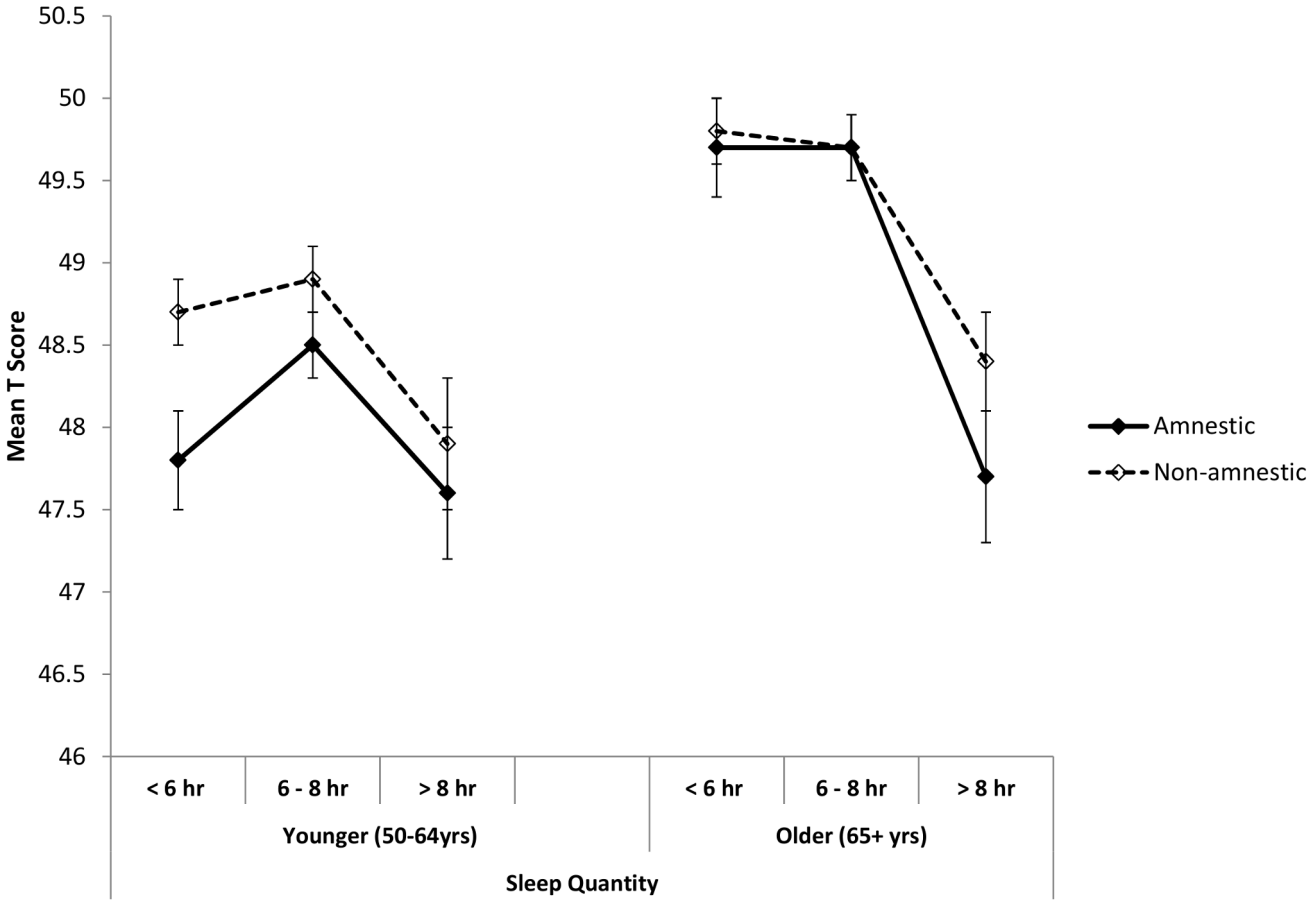
Cognitive function scores by sleep quantity. Fully adjusted mean T scores for amnestic and non-amnestic cognition scores for each sleep quantity category, in younger and older age groups. Adjusted for age, sex, sleep*age, education, employment grade, depression, physical activity, smoking, general health (Model 3).

**Figure 2:**
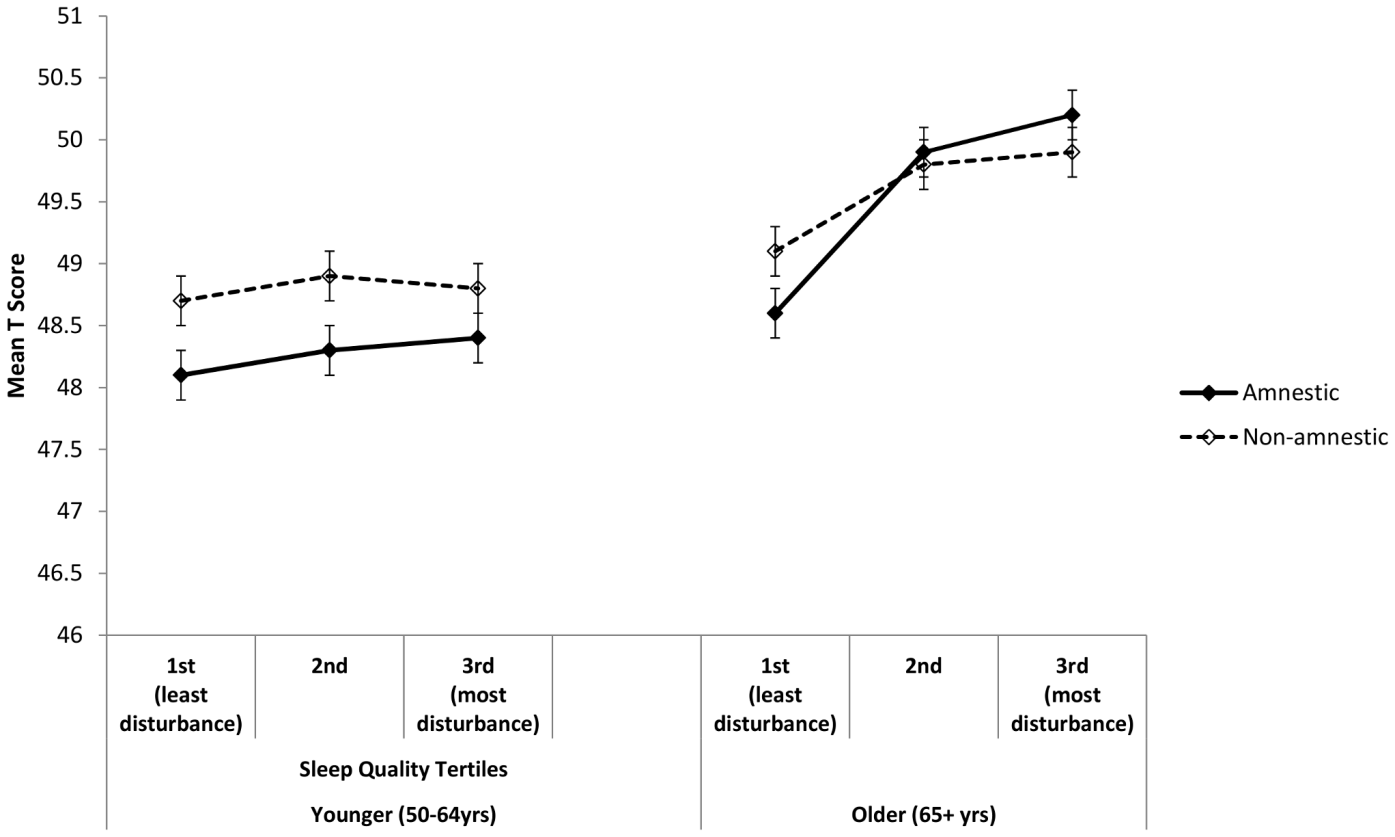
Cognitive function scores by sleep quality. Fully adjusted mean T scores for amnestic and non-amnestic cognition scores for each sleep quality tertile, in younger and older age groups. Adjusted for age, sex, sleep*age, education, employment grade, depression, physical activity, smoking, general health (Model 3).

After adjustment for multiple confounders, there was a statistically significant association between sleep quantity (Table 3 and Figure 1) and both amnestic and non-amnestic cognitive function (mean T score) in both age groups. In a fully adjusted model (Model 3), in the younger group duration of sleep explained 15.2% of the variance in the amnestic domain (p = 0.003) and 20.6% of non-amnestic cognitive function (p = 0.010). In the older group the estimates were 21.3% (p<0.001) and 25.6% (p<0.001), respectively. Applying the same adjustments to sleep quality, there was a statistically significant association between sleep quality and both amnestic (p<0.001) and non-amnestic (p<0.001) cognition in the older age group, but no longer in the younger age group (p = 0.586 and p = 0.373, respectively; Table 4). Again, sleep quality explained between 15.1% and 25.5% of the variance in the cognition domains (Table 4 and Figure 2). When these analyses were carried out after the exclusion of participants with previous stroke, Parkinson’s, Alzheimer’s and dementia or memory impairment (n = 419) the results did not change substantially (data not shown).

**Table 3:** Mean amnestic and non-amnestic T scores by sleep quantity categories for the younger and older age groups.

AMNESTIC		YOUNGER GROUP (50–64 yrs)		OLDER GROUP (65+yrs)	
Model	Sleep Quantity	Mean T Score(SE)	*p* and R^2^ values	Mean T Score(SE)	*p* and R^2^ values
**0**	<6 hr	48.4 (0.2)		49.4 (0.3)	
*(unadjusted)*	6–8 hr	50.4 (0.1)		50.4 (0.1)	
	>8 hr	48.6 (0.4)	*p*<0.001; R^2^ = 0.014	47.5 (0.4)	*p*<0.001; R^2^ = 0.016
**1**	<6 hr	48.3 (0.2)		49.4 (0.3)	
(age+sex, sleep*age)	6–8 hr	50.3 (0.1)		50.4 (0.1)	
	>8 hr	48.6 (0.4)	*p*<0.001; R^2^ = 0.028	48.1 (0.3)	*p*<0.001; R^2^ = 0.125
**2**	<6 hr	48.5 (0.2)		50.0 (0.3)	
*(Model 1+ education,*	6–8 hr	49.8 (0.1)		50.5 (0.1)	
*employment grade)*	>8 hr	48.7 (0.4)	*p*<0.001; R^2^ = 0.123	48.4 (0.3)	*p*<0.001; R^2^ = 0.189
**3**	<6 hr	47.8 (0.3)		49.7 (0.3)	
*(Model 2+ depression, physical,*	6–8 hr	48.5 (0.2)		49.7 (0.2)	
*activity, smoking, general health)*	>8 hr	47.6 (0.4)	*p* = 0.003; R^2^ = 0.152	47.7 (0.4)	*p*<0.001; R^2^ = 0.213
**NON-AMNESTIC**					
**0**	<6 hr	48.8 (0.2)		49.2 (0.2)	
*(unadjusted)*	6–8 hr	50.3 (0.1)		50.4 (0.1)	
	>8 hr	48.4 (0.4)	*p*<0.001; R^2^ = 0.014	48.3 (0.3)	*p*<0.001; R^2^ = 0.013
**1**	<6 hr	48.8 (0.2)		49.4 (0.2)	
(age+sex, sleep*age)	6–8 hr	50.3 (0.1)		50.3 (0.1)	
	>8 hr	48.5 (0.4)	*p*<0.001; R^2^ = 0.023	48.7 (0.3)	*p*<0.001; R^2^ = 0.110
**2**	<6 hr	49.0 (0.2)		50.1 (0.2)	
*(Model 1+ education,*	6–8 hr	49.7 (0.1)		50.5 (0.1)	
*employment grade)*	>8 hr	48.5 (0.3)	*p*<0.001; R^2^ = 0.182	49.1 (0.3)	*p*<0.001; R^2^ = 0.225
**3**	<6 hr	48.7 (0.2)		49.8 (0.2)	
*(Model 2+ depression, physical,*	6–8 hr	48.9 (0.2)		49.7 (0.2)	
*activity, smoking, general health)*	>8 hr	47.9 (0.4)	*p* = 0.010; R^2^ = 0.206	48.4 (0.3)	*p*<0.001; R^2^ = 0.256

**Table 4:** Mean amnestic and non-amnestic T scores by sleep quality categories for the younger and older age groups.

AMNESTIC		YOUNGER GROUP (50–64yrs)		OLDER GROUP (65+yrs)	
Model	Sleep Quality	Mean TScore (SE)	*p* and R^2^ values	Mean TScore (SE)	*p* and R^2^ values
**0**	1st tertile (least disturbance)	50.3 (0.2)		49.7 (0.2)	
*(unadjusted)*	2nd tertile	50.2 (0.2)		50.5 (0.2)	
	3rd tertile (most disturbance)	49.4 (0.2)	*p*<0.001; R^2^ = 0.005	49.8 (0.2)	*p* = 0.002; R^2^ = 0.003
**1**	1st tertile (least disturbance)	50.4 (0.2)		49.6 (0.2)	
(age+sex, sleep*age)	2nd tertile	50.2 (0.2)		50.5 (0.2)	
	3rd tertile (most disturbance)	49.1 (0.2)	*p*<0.001; R^2^ = 0.020	49.7 (0.2)	*p*<0.001; R^2^ = 0.114
**2**	1st tertile (least disturbance)	49.8 (0.2)		49.8 (0.2)	
*(Model 1+ education,*	2nd tertile	49.7 (0.2)		50.7 (0.2)	
*employment grade)*	3rd tertile (most disturbance)	49.1 (0.2)	*p* = 0.005; R^2^ = 0.119	50.2 (0.2)	*p* = 0.002; R^2^ = 0.181
**3**	1st tertile (least disturbance)	48.1 (0.2)		48.6 (0.2)	
*(Model 2+ depression, physical*	2nd tertile	48.3 (0.2)		49.9 (0.2)	
*activity, smoking, general health)*	3rd tertile (most disturbance)	48.4 (0.2)	*p* = 0.586; R^2^ = 0.151	50.2 (0.2)	*p*<0.001; R^2^ = 0.213
**NON-AMNESTIC**					
**0**	1st tertile (least disturbance)	50.5 (0.1)		50.1 (0.2)	
*(unadjusted)*	2nd tertile	50.3 (0.1)		50.4 (0.2)	
	3rd tertile (most disturbance)	49.1 (0.1)	*p*<0.001; R^2^ = 0.011	49.4 (0.2)	*p*<0.001; R^2^ = 0.005
**1**	1st tertile (least disturbance)	50.4 (0.1)		50.1 (0.1)	
(age+sex, sleep*age)	2nd tertile	50.4 (0.1)		50.4 (0.2)	
	3rd tertile (most disturbance)	49.2 (0.1)	*p*<0.001; R^2^ = 0.020	49.5 (0.2)	*p*<0.001; R^2^ = 0.105
**2**	1st tertile (least disturbance)	49.7 (0.1)		50.3 (0.1)	
*(Model 1+ education,*	2nd tertile	49.8 (0.1)		50.6 (0.1)	
*employment grade)*	3rd tertile (most disturbance)	49.1 (0.1)	*p* = 0.001; R^2^ = 0.181	50.1 (0.2)	*p* = 0.067; R^2^ = 0.221
**3**	1st tertile (least disturbance)	48.7 (0.2)		49.1 (0.2)	
*(Model 2+ depression, physical*	2nd tertile	48.9 (0.2)		49.8 (0.2)	
*activity, smoking, general health)*	3rd tertile (most disturbance)	48.8 (0.2)	*p* = 0.373; R^2^ = 0.204	49.9 (0.2)	*p*<0.001; R^2^ = 0.255

#### Sleep quantity and amnestic function in younger group (Figure 1)

After Bonferroni correction short sleepers had significantly lower mean amnestic T scores than optimal sleepers (*p* = 0.012). There was no significant difference in amnestic scores between short sleepers and long sleepers (*p*>0.999), or between optimal sleepers and long sleepers (*p* = 0.088).

#### Sleep quantity and amnestic function in older group (Figure 1)

Long sleepers had significantly lower amnestic scores than both short sleepers (*p*<0.001) and optimal sleepers (*p*<0.001), whereas there was no significant difference between short and optimal sleepers (*p*>0.999).

#### Sleep quantity and non-amnestic function in younger group (Figure 1)

Long sleepers had significantly lower mean non-amnestic T scores than optimal sleepers (*p* = 0.010), but there was no significant difference in non-amnestic T scores between short sleepers and optimal sleepers (*p* = 0.789), or short sleepers and long sleepers (*p* = 0.161).

#### Sleep quantity and non-amnestic function in older group (Figure 1)

Long sleepers had significantly lower non-amnestic scores than both short sleepers (*p*<0.001) and optimal sleepers (*p*<0.001), but there was no significant difference in non-amnestic scores between short and optimal sleepers (*p*>0.999).

#### Sleep quality and amnestic function in younger group (Figure 2)

There were no significant differences in amnestic scores between any of the sleep quality tertiles (all *p*>0.917).

#### Sleep quality and amnestic function in older group (Figure 2)

Those reporting the least amount of sleep disturbance had the lowest cognitive function scores. Mean T scores were significantly lower in the 1^st^ tertile (least disturbance) than in the 2^nd^ and 3^rd^ tertiles (both *p*<0.001), but the difference between 2^nd^ and 3^rd^ tertile was not significant (*p* = 0.461).

#### Sleep quality and non-amnestic function in younger group (Figure 2)

There were no significant differences in non-amnestic scores between any of the sleep quality tertiles for the younger age group (all *p*>0.484).

#### Sleep quality and non-amnestic function in older group (Figure 2)

Those in the 1^st^ tertile (least disturbance) had lower T scores than those in the 2^nd^ (*p* = 0.003) or 3^rd^ (*p* = 0.001) tertile, but there was no significant difference in T scores between the 2^nd^ and 3^rd^ tertile (*p*>0.999).

### Interaction of sleep quality with age on cognitive function

**Figure 3:**
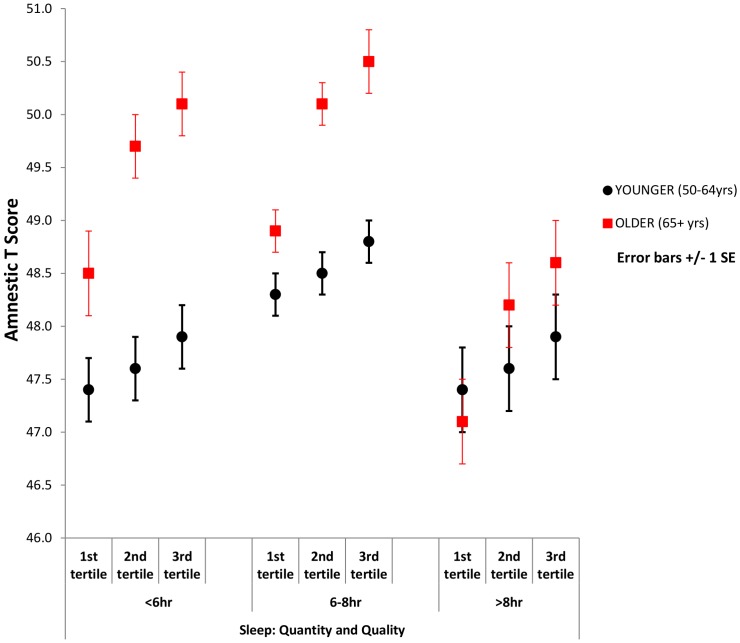
Mean amnestic T scores by sleep categories adjusted for sleep quality. Adjusted mean amnestic T Score by sleep quantity categories, per sleep quality tertile, in younger and older age groups.

**Figure 4:**
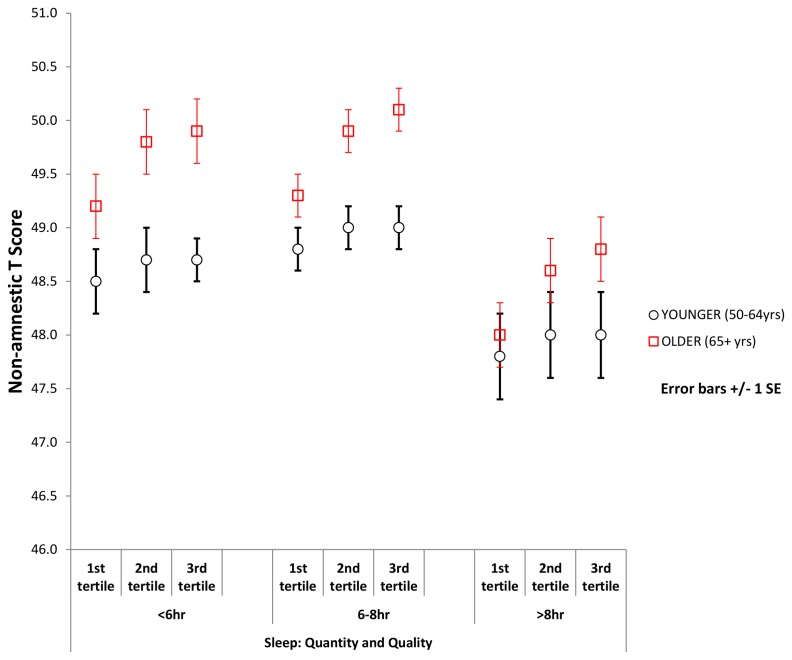
Mean non-amnestic T scores by sleep categories adjusted for sleep quality. Adjusted mean non-amnestic T Score by sleep quantity categories, per sleep quality tertile, in younger and older age groups.

We detected a significant interaction between sleep quality and age independent from sleep quantity, more evident for the association with amnestic cognitive function (see above). These interactions are shown in Figure 3 for amnestic function and Figure 4 for non-amnestic function when stratified by sleep duration. At any level of sleep duration there was a steeper association between sleep quality and amnestic cognition in the older than the younger group.

## Discussion

### Main Findings

The results from this study suggest that sleep quantity and quality are associated with both amnestic and non-amnestic cognition, and that these associations differ with age. Following adjustment for potential confounders, in the younger age group (50–64 years), both short (<6 hrs per night) and long (>8 hrs per night) sleep were associated with lower amnestic and non-amnestic scores. Whereas in the older age group (65+ years), associations were only significant with long sleep. However, whilst sleep quality was associated with both amnestic and non-amnestic scores in the older age group, it was not in the younger age group. These effects were regardless of duration of sleep. Finally, the older individuals with higher cognitive function reported more often sleep disturbances, regardless of sleep quantity.

### External validity and comparison with other studies

Previous cross-sectional studies have demonstrated that long sleep durations are associated with diminished global cognition [33]. Furthermore, in older populations there may be an inverse U-shaped relationship between sleep and cognition, with both short and long sleep being associated with poorer memory function [11].

In our analysis we separated the individuals into younger and older groups, to examine the potential differences in the associations between sleep and cognition in these two age groups. In agreement with Faubel et al. [33] who showed an association between long sleep and diminished global cognition, we found a significant decrease in amnestic and non-amnestic cognitive function in long sleepers, but this only reached significance in the older group. In our younger group, amnestic scores were significantly lower in short sleepers, whereas non-amnestic scores were lower in long sleepers. Our results also support those of Xu et al. [11], who suggested that memory function is impaired in short and long sleepers. Again however, we have explored the effect of sleep on memory in further detail by examining this in different pre- and post-retirement age groups. Our results show that an inverted U-shaped relationship exists in younger adults, where the amnestic scores for short sleepers were significantly lower than those for optimal sleepers, and whilst the amnestic scores for the long sleepers were reduced, this difference was not statistically significant. These findings could be interpreted in the context of recent findings in mice, which suggest that sleep deprivation causes irreversible damage to the brain which could impair cognitive function, particularly alertness [34]. However, if this is the case, it is not clear why the effect of short sleep is not evident in the older group. That is, in the older adults, there was no observed effect in short sleepers but the amnestic scores in long sleepers were significantly lower than those for optimal sleepers. These findings are consistent with the results described in a recent cross-sectional analysis of an elderly population [35]. Using the MMSE long, but not short, sleep was associated with significantly lower cognitive function scores. Furthermore, whilst another cross-sectional study reported that both short and long sleep durations were associated with diminished global cognition, the individuals in that study were aged 30 years or older and hence included both ‘much younger’ and ‘older’ adults [10].

Our results indicate that the age of the population under investigation may be an important determinant of study outcome. In ELSA, those over the age of 89 years are coded with an age of 99 years to promote anonymity within the sample, and as such, the coded age is not accurate for individuals over the age of 89 years. Therefore, the age range in our sample was 50–89 years. It may be pertinent for future studies to examine the association between sleep and cognition and the influence of extraneous health and lifestyle factors, in the very old (i.e. those over the age of 90 years) to give a more comprehensive and inclusive overview of the cognitive ageing process.

In this study, we did not find any significant interaction with gender (interactions between sleep and sex factors in Model 3 for younger and older groups, all *p*≥0.085) and hence the data was not analysed in men and women separately. However, it is of interest to note that it has been suggested [36] that whereas women have higher baseline cognition scores than men, decline may be faster in women, and so longitudinal trajectories of male and female cognitive decline converge in later life. This could explain the lack of sex interactions in our results, but it is important to note for future studies of younger adult populations.

In a study in men only, Blackwell et al. [15] suggested that disturbed sleep is strongly associated with decline in executive function (or non-amnestic function), and less so for global cognition, whereas we found the opposite to be true in older adults. Indeed in our older group, the highest cognitive function scores (both amnestic and non-amnestic) were seen in those individuals with the greatest reported disturbances in sleep. In younger individuals however, there was no significant association between cognition and sleep quality, indicating that until we reach the age of around 65 years, there may be no association between sleep quality and cognitive function. The reason for these differences is unclear and prospective analyses of the effects of sleep quality on the decline in cognition could help rule out possible influences of reverse causality due to pre-existing ill-health or other confounders.

The suggestion that cognitive function increases with increasing sleep disturbance in older individuals appears to be counterintuitive. There are a number of possible reasons for this that need to be explored. Higher cognitive scores in older individuals could be due to practice effects – i.e. the older group may have more experience with the cognitive function tests in ELSA [37]. It may reflect the fact that those individuals who are more cognitively able are better at recording sleep disturbance data. Alternatively, it may indicate that in an elderly population, individuals who are more cognitively active may process the day’s events and/or experience more worry or anxiety than those who are less cognitively active, and hence this may lead to an associated increase in self-reported frequency of sleep disturbance. Further, previous research has indicated that mild anxiety symptoms in older adults are associated with better cognitive function [38], which would support this suggestion. Confounding effects of medications may also be more important in an older group. Likewise, in those participants with memory problems, we cannot exclude the possibility that their responses might have been erroneous to some extent due to their memory impairment. The number of such individuals was small and their exclusion did not substantially change the results. Some antihypertensives and corticosteroids acts as stimulants and, the night-time use of diuretics can promote repeated awakening to go to the bathroom. These ideas and the temporal sequence of events, however, need to be addressed in longitudinal studies.

A small number of prospective studies have investigated whether poor sleep can *predict* cognitive impairment in later life, but these have produced inconsistent findings [39]–[43]. The heterogeneity of results between these studies could be due to a number of methodological differences, including age [14], [26] and sex [3], [44] of participants, duration of follow-up [41]–[43], population culture or ethnicity [45], [46], cognitive assessments or sleep measures [41], [47], and statistical adjustments made for various potential confounders. There are two recent prospective studies, which are most pertinent to our line of enquiry [14], [26]. The Whitehall II study shows that adverse changes in sleep quantity over time (either a decrease from 6, 7 or 8 hours, or an increase from 7 or 8 hours, over a mean period of 5.4 years) are associated with lower scores on a variety of tests of cognitive function, with the exception of memory tasks [26]. Thus, detrimental changes in sleep quantity over time, particularly a shift to longer sleep durations, may have a domain-specific effect on non-amnestic cognitive performance, whilst memory function may remain relatively preserved. This study was carried out in a middle-aged cohort. Whilst it remains to be seen whether changes in sleep duration would have similar effects on different cognitive domains in older adults one report suggests a detectable effect on verbal memory in participants >70 years of age [48].

It is of interest that alcohol and quality of life scores were associated with an attenuation of the association between sleep and cognition. This may be due to protective effects of alcohol [49] and quality of life [50] on cognitive function.

### Strengths and limitations

Whilst in a recent study it was demonstrated that the MMSE can be used as a tool to extract scores pertaining to amnestic and non-amnestic function specifically [14], the findings should be interpreted with caution. That is, reliance on scores from one item of a test of global cognition is not a robust method of diagnosing or even suggesting the existence of a memory impairment - not merely because there are so many more tests which comprise the non-amnestic score on the MMSE. A major strength of our study is that the cognitive function assessments are robust and have been used extensively in previous population studies, such as the Medical Research Council Cognitive Function and Aging Study (MRC CFAS) [51], Health and Retirement Study (HRS) [52], and the MRC National Survey of Health and Development (also known as the British 1946 birth cohort) [53]. Furthermore, the amnestic and non-amnestic composite scores used in our analyses were derived from multiple tests, thus minimising ceiling and floor effects.

Limitations of this study include the exclusion of non-white individuals due to the limited sample size (3.5%) and hence lack of statistical power for the analysis, the assessment of sleep domains based exclusively on self-reporting and the cross-sectional design. The latter precludes investigation of the temporal sequence of sleep disturbances in relation to cognitive function. This will be addressed in future analyses when further waves of ELSA follow-up data are available. Repeated measures in longitudinal studies can however introduce a possible practice effect which masks cognitive decline over time [37]. Finally, data on the use of hypnotics was not collected in ELSA and hence we were unable to adjust for a potential important confounder.

Some studies have categorised short sleep as <5 hrs and long sleep as >9 hrs [14], [26], but this was not possible for our analyses, owing to the very small numbers in these groups and subsequent loss of statistical power. Nevertheless, it is widely accepted that around 7 hrs of sleep per night is considered to be optimal [23], and so our sleep quantity categories are generally consistent with, and comparable to, the majority of studies in this field.

### Implications

The results from this study suggest that sleep quantity and sleep quality, if causally related to cognition, may have different effects in younger and older adults, on both amnestic and non-amnestic cognitive function. Furthermore, there are clear interactions between sleep quantity and sleep quality with age, which would indicate that future studies need to be designed to investigate both the independent effects, and interplay between, these different sleep measures throughout the life-course.

### Conclusions

Ideally, the diagnosis of mild cognitive impairment needs to be made at a very early stage, where intervention might delay or even prevent the disease process and potential progression to various forms of dementia. Further research is needed, however, to investigate the temporal sequence of events underlying the association between sleep quantity and quality and cognitive function and decline over time. Furthermore, it is important that studies develop validated and standardised tests, which are specifically designed to detect amnestic and non-amnestic cognitive impairments in the normal ageing population. This would allow direct comparison between studies with different populations, and in in particular, those with differing age groups.

## Supporting Information

Figure S1 **Sleep quantity and sleep quality.** Mean sleep quality score for each sleep quantity category (upper panel, S1A-S1B), and mean sleep quantity (hours) for each sleep quality tertile (lower panel, S1C-S1D), in younger and older age groups. All unadjusted ANOVAs *p*<0.001; see figures for *p* values for multiple comparisons (Bonferroni-corrected).
(TIF)
